# Heritable alteration in salt-tolerance in rice induced by introgression from wild rice (*Zizania latifolia*)

**DOI:** 10.1186/1939-8433-5-36

**Published:** 2012-12-19

**Authors:** Chunwu Yang, Tianyuan Zhang, Huan Wang, Na Zhao, Bao Liu

**Affiliations:** Key laboratory of Molecular Epigenetics of MOE, Northeast Normal University, Changchun, 130024 China; Department of Agronomy, Jilin Agricultural University, Changchun, 130118 China

## Abstract

**Background:**

Introgression as a means of generating phenotypic novelty, including altered stress tolerance, is increasingly being recognized as common. The underlying basis for *de novo* genesis of phenotypic variation in the introgression lines remains largely unexplored. In this investigation, we used a rice line (*RZ35*) derived from introgressive hybridization between rice (*Oryza sativa* L.) and wild rice (*Zizania latifolia* Griseb.), along with its rice parental line (cv. *Matsumae*) as the experimental materials. We compared effects of salt stress on growth, ion homeostasis, and relevant gene expression between *RZ35* and *Matsumae*, to explore possible mechanisms of heritable alteration in stress tolerance induced by the introgression.

**Results:**

Contrary to our expectation, the results showed that the inhibitory effect of salt stress on growth of *RZ35* was significantly greater than that of *Matsumae*. We further found that a major underlying cause for this outcome is that the introgression process weakened the capacity in Na^+^ exclusion under the salt stress condition, and hence, escalated the injuries of Na^+^ and Cl^-^ in shoots of *RZ35*. Accordingly, based on q-RT-PCR analysis, four genes known to be involved in the Na^+^ exclusion, i.e., *OsHKT1;5*, *OsSOS1*, *OsCIPK24* and *OsCBL4*, were found to be significantly down-regulated in roots of *RZ35* relative to its rice parental line under the salt stress condition, thus implicating a gene expression regulation-based molecular mechanism underlying the difference in salt stress-tolerance between the introgression line and its rice parental line.

**Conclusions:**

We show that introgression represents a potent means for rapidly generating *de novo* heritable variations in physiological traits like stress tolerance in plants, although the direction of the alteration appears unpredictable.

**Electronic supplementary material:**

The online version of this article (doi:10.1186/1939-8433-5-36) contains supplementary material, which is available to authorized users.

## Background

Interspecific hybridization plays a pervasive role in genome evolution through the formation of homoploid hybrids, allopolyploids and introgressants (Ainouche et al. 2004; Hochholdinger and Hoecker 2007; Lippman and Zamir 2007; Soltis and Soltis 2009). Introgressive hybridization once being considered as “unsuccessful hybridization” turned out as frequent incidents in natural plant populations (Arnold 2004). Introgression has the potential to allow adaptation to evolve at rates that may considerably exceed those possible for non-hybridizing populations that are dependent on random mutation for genetic novelty (Barton 2001). For example, Whitney *et al.* (2010) demonstrated that introgression had altered multiple aspects of the *Helianthus annuus* phenotypes in an adaptive manner, affected traits relevant to adaptation to both biotic and abiotic environments (Whitney et al. 2010). In plant breeding, introgression of uncharacterized DNA segments from a wild species into a cultivated variety is a commonly used approach. Intriguingly, it is recently recognized that introgression not only produces novel traits via the expected transfer from one parental species to another and genic interaction between parental genomes, but may also generate *de novo* genetic and epigenetic variations (Ainouche et al. 2004; Wang et al. 2005; Chapman and Abbott 2010). Nevertheless, the underlying basis for the *de novo* genesis of heritable phenotypic variation via introgression remains largely unknown (Baack and Rieseberg 2007; Chapman and Abbott 2010).

Under natural habitat, plants are constantly exposed to biotic or abiotic stress conditions such as pathogen and insect infestation, drought, salinity, and high- or low temperatures. Of these stress conditions, salinity is a widespread environmental problem and an important factor limiting agricultural productivity of many crops. In this study, we chose a rice line derived from introgressive hybridization with wild rice (*Zizania latifolia* Griseb.) as the experimental material. *Z. latifolia* grows luxuriantly around paddy fields, and represents a potentially valuable tertiary gene pool for rice improvement (Abedinia et al. 2000). Although the two species are sexually incompatible (Abedinia et al. 2000), we have successfully constructed a set of rice introgressants containing only minute amounts of *Z. latifolia* genomic DNA by a novel sexual hybridization approach (Liu et al. 1999; Shan et al. 2005). Using both amplified fragment length polymorphism (AFLP) analysis (Wang et al. 2005) and gel-blotting (Shan et al. 2005), we documented presence of *Zizania* species-specific DNA segments in the introgression lines including the one (RZ35) used in this study. Moreover, we showed that these introgression lines contained an array of genetic and epigenetic variations that occurred *de novo* including rampant mobilization of several transposable elements endogenous to the rice genome (Liu et al. 2004; Shan et al. 2005; Wang et al. 2005). Accordingly, field tests showed that the introgressants exhibited heritable and novel morphological characteristics in multiple traits compared with their rice parental cultivar *Matsumae* (Shan et al. 2005; Wang et al. 2005). Of these introgressants, one (named *RZ35*) is of exceptional interest as it exhibited multiple novel phenotypic traits compared with its rice parental line *Matsumae*. For example, compared with *Matsumae*, *RZ35* showed changes in nitrogen use efficiency, prolonged phenophase, and enhanced resistance to the blast disease (Shan et al. 2005; Wang et al. 2005). However, the physiological and molecular mechanisms of these interesting phenotypic alterations remained unknown.

Salt stress in soil generally involves osmotic stress and ion-induced injury, and Na^+^ is the main toxic ion in salinized soil. The extent of tolerance by plants to Na^+^ stress depends on at least three processes: compartmentalization (at cellular and/or tissue levels), exclusion (from roots into the rhizosphere) and transportation (in vasculatures) of the ions. In *Arabidopsis*, the salt overly sensitive protein 1 (SOS1) functions in Na^+^ exclusion from root epidermal cells into the rhizosphere, which also plays a role in retrieving Na^+^ from the xylem stream under severe salt stress (Shi et al. 2002). The Ca^2+^-responsive AtSOS3–AtSOS2 (AtCIPK24-AtCBL4) protein kinase pathway mediates regulation of the expression and activities of Na^+^ transporters such as AtSOS1 and AtNHX, a Na^+^/H^+^ exchanger that mediates Na^+^ compartmentalization into vacuoles (Zhu 2003). The rice SOS salt tolerance pathway has been identified and its functions have been shown as similar to that of the SOS pathway in *Arabidopsis* (Martínez-Atienza et al. 2007). In *Arabidopsis* and some other plant species, the Na^+^/H^+^ exchanger (NHX) family has been shown to function in Na^+^ compartmentalization into vacuoles (Munns and Tester 2008). In addition, some members of the high affinity K^+^ transporter (HKT) family, such as OsHKT1;5 and AtHKT1;1, mediate Na^+^ exclusion from shoots via Na^+^ removal from the xylem sap (Horie et al. 2009; Negrão et al. 2011).

Given the previously observed multiple phenotypic variations in the introgression line *RZ35*, describe above, we were interested to explore whether a particular physiological trait, i.e., tolerance to salinity, was altered in this line relative to its rice parental line (*Matsumae*). For this purpose, we compared effects of salt stress on growth, ion content, and expression level of several genes related to K^+^/Na^+^ metabolism between *RZ35* and *Matsumae*. We found that relative to *Matsumae*, the salt tolerance trait in RZ5 was significantly compromised due to weakened capacity in Na^+^ exclusion under the salt stress condition, and hence, escalated the injuries of Na^+^ and Cl^-^ in shoots. Accordingly, four genes known to be involved in the Na^+^ exclusion, i.e., *OsHKT1;5*, *OsSOS1*, *OsCIPK24* and *OsCBL4*, were found to be significantly down-regulated in roots of *RZ35* relative to its rice parental line under the salt stress condition, thus implicating a gene expression regulation-based molecular mechanism underlying the difference in salt tolerance between the introgression line and its rice parental line.

## Results

### The Tolerance to Salt Stress Was Compromised in Introgression Line *RZ35* Relative to Its Rice Parental Line *Matsumae*

**Figure 1:**
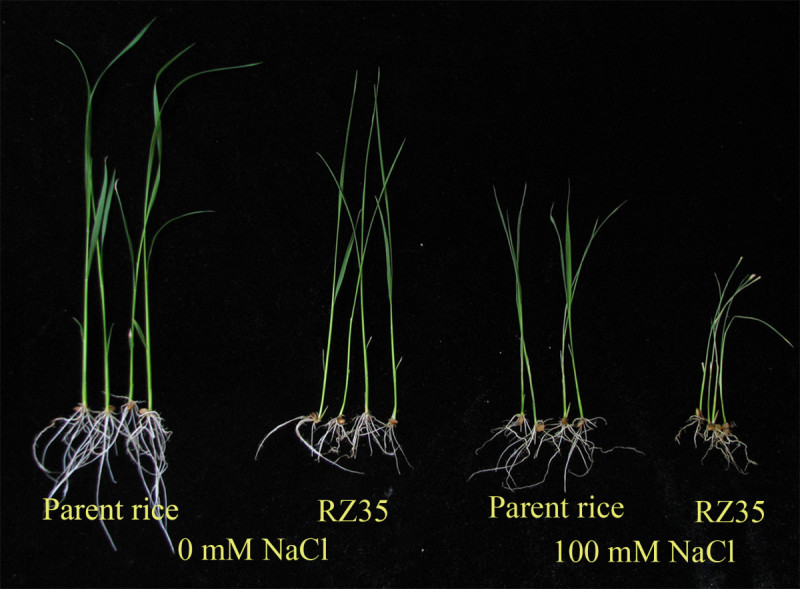
**Effects of salt stress on growth in the introgression line** ***RZ35*** **and its rice parental line,** ***Matsumae*** **.** 14-day-old seedlings were subjected to salt stress (100 mM NaCl) for 5 d. The values shown are the means (± SE) of three biological replicates. Different letters above bars represent significantly different, according to least significant difference (LSD) test (*P* <0.05).

**Figure 2:**
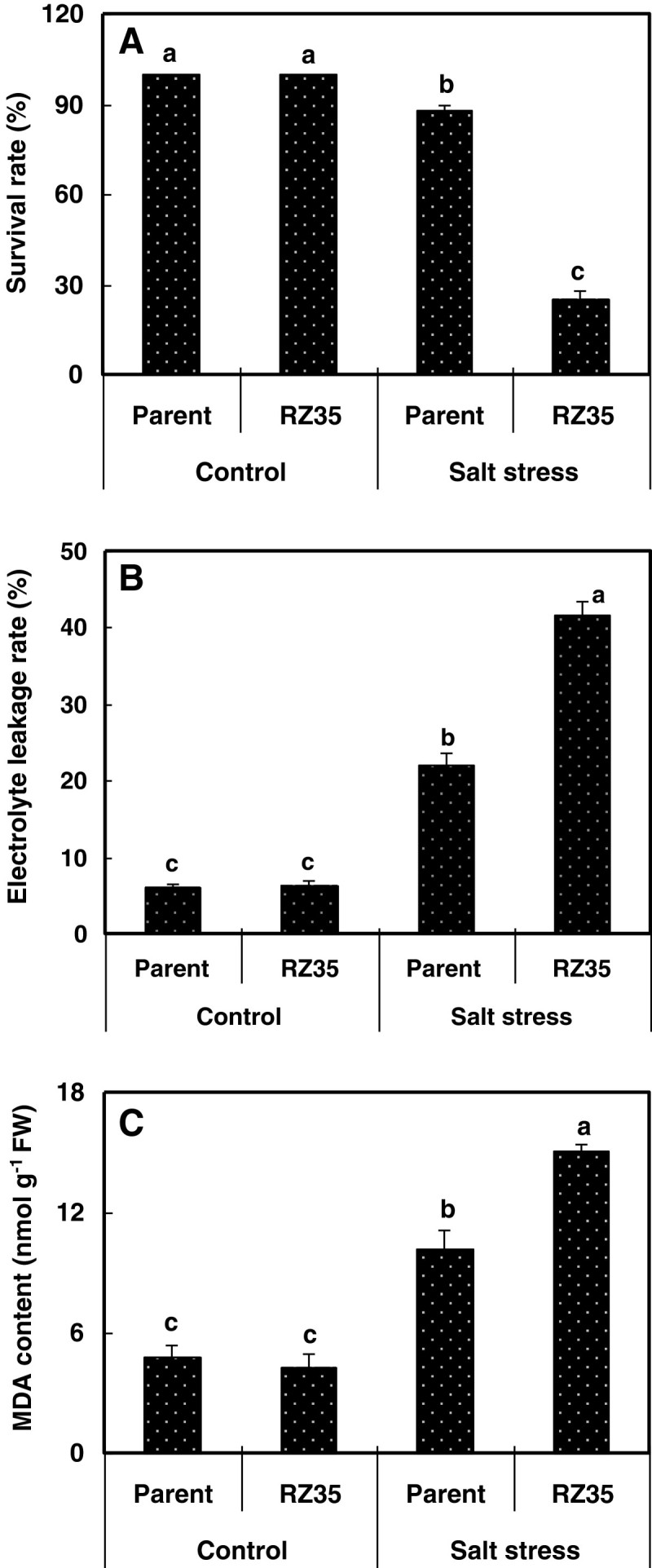
**Effects of salt stress on survival rate, MDA (malondialdehyde) content and electrolyte leakage rate in the introgression line** ***RZ35*** **and its rice parental line,** ***Matsumae*** **.** 14-day-old seedlings were subjected to salt stress (100 mM NaCl). When the seedlings were subjected to salt stress for 5 d, the MDA content and electrolyte leakage rate of shoots were mensurated. When the seedlings were subjected to salt stress for 13 d, the survival rates were calculated. The values shown are the means (± SE) of three biological replicates. Different letters above bars represent significantly different, according to least significant difference (LSD) test (*P* <0.05).

Figure 1 shows that the inhibitory effect of salt stress on growth of the introgression line *RZ35* was greater than that of *Matsumae* at the seedling stage. Furthermore, a survivorship assay showed that under unstressed control condition, both lines had 100% survival rate, while under salt stress condition (100 mM NaCl for 13d), the survival rate of *RZ35* was only 25.3%, and that of *Matsumae* was 88% (Figure 2). The membrane damage of salt stress could be reflected by the MDA (malondialdehyde) content, with a higher MDA content denoting a stronger harm of salt stress of the membrane. Although the salt stress increased the electrolyte leakage rates and MDA contents of both lines, the values of *RZ35* were significantly higher than *Matsumae* under salt stress (Figure 2, *P<* 0.05). These results established that the salt tolerance of *RZ35* was lower than *Matsumae* at the seedling stage.

### Differential Accumulation of Ions between Introgression Line *RZ35* and Its Rice Parental Line *Matsumae*

**Figure 3:**
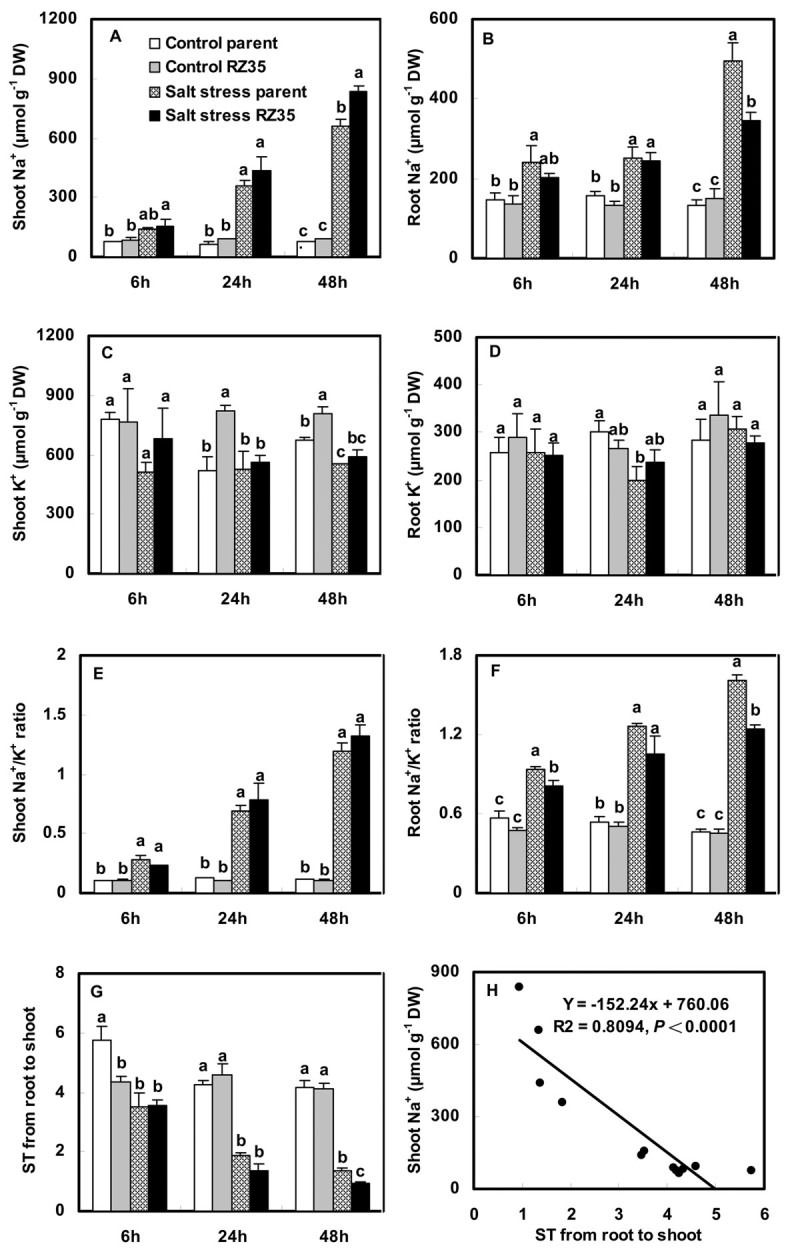
**Effects of salt stress on Na**^**+**^**, K**^**+**^**, Na**^**+**^**/K**^**+**^**and ST in the introgression line** ***RZ35*** **and its rice parental line,** ***Matsumae*** **.** The values shown are the means (± SE) of three biological replicates. Means followed by different letters among treatments at same time point are significantly different, according to least significant difference (LSD) test (*P* <0.05). 14-day-old seedlings were subjected to salt stress (100 mM NaCl) for 6 h, 24 h and 48 h. ST, selective transport capacity by different parts of plant for K^+^ over Na^+^. ST, selective transport capacity by different parts of plant for K^+^ over Na^+^. ST (root/shoot) = (Na^+^/K^+^ in root)/(Na^+^/K^+^ in shoot). The correlation analysis between ST and shoot Na^+^ content was performed (**H**).

**Figure 4:**
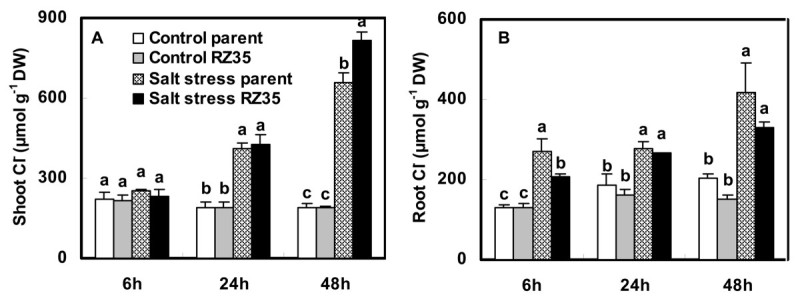
**Effects of salt stress on Cl**^**-**^**contents in the introgression line** ***RZ35*** **and its rice parental line,** ***Matsumae*** **.** The values shown are the means (± SE) of three biological replicates. Means followed by different letters among treatments at same time point are significantly different, according to least significant difference (LSD) test (*P* <0.05). 14-day-old seedlings were subjected to salt stress (100 mM NaCl) for 6 h, 24 h and 48 h.

Salt stress increased contents of Na^+^ and Cl^-^ and Na^+^/K^+^ ratio, and decreased content of K^+^ (Figures 3 and 4). When the rice seedlings were subjected to salt stress for 6 h and 24 h, the Na^+^, Na^+^/K^+^ and Cl^-^ of *RZ35* were similar to *Matsumae*. However, at 48 h of salt stress, the Na^+^, Na^+^/K^+^, and Cl^-^ in shoots of *RZ35* were higher than those of *Matsumae* (Figures 3 and 4, *P*<0.05), while their values in roots of *RZ35* were lower than those of *Matsumae* (Figures 3 and 4, *P*<0.05). The difference between the two lines for K^+^ accumulation was minimal and insignificant (Figures 3C-D). A higher selective transport capacity (ST) indicated stronger capacity by roots to control the transportation of Na^+^ from root to shoot. The ST value was correlated negatively with the Na^+^ content in rice shoots (Figure 3H, *P*<0.0001). The ST value of *RZ35* was lower than *Matsumae* at 48 h under salt stress (Figure 3G, *P*<0.05).

### Altered Expression of the Salt Overly Sensitive (SOS) Pathway and the *Na*^*+*^*/H*^*+*^*Exchanger (NHX)* Gene Families in Introgression Line *RZ35*

**Figure 5:**
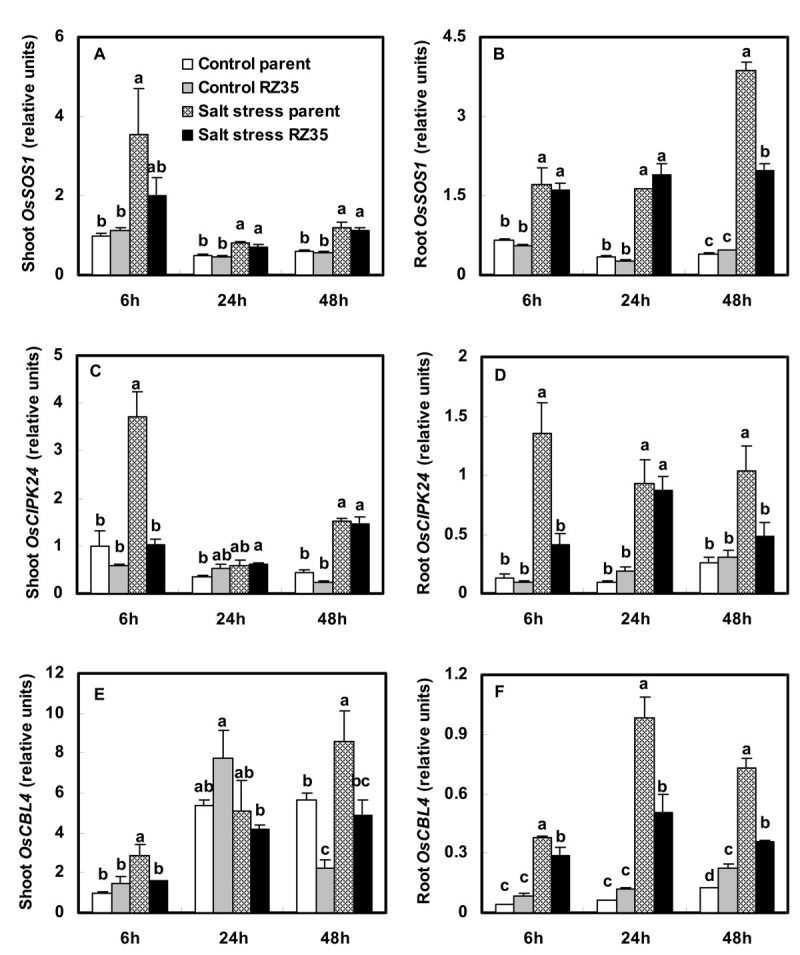
**Effects of salt stress on the expression of** ***OsSOS1*** **,** ***OsCIPK24*** **and** ***OsCBL4*** **in the introgression line** ***RZ35*** **and its rice parental line,** ***Matsumae*** **.** The values shown are the means (± SE) of three biological replicates. Means followed by different letters among treatments at same time point are significantly different, according to least significant difference (LSD) test (*P* <0.05). 14-day-old seedlings were subjected to salt stress (100 mM NaCl) for 6 h, 24 h and 48 h.

**Figure 6:**
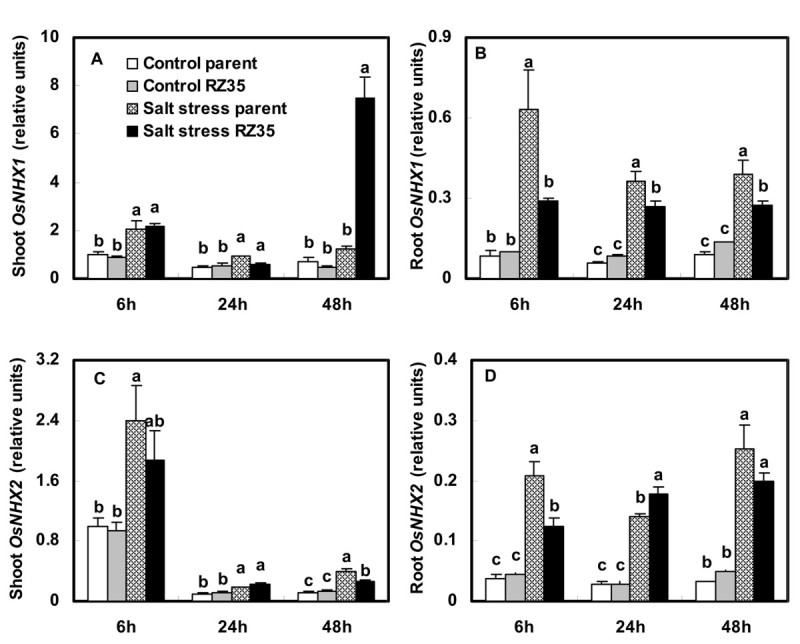
**Effects of salt stress on the expression of** ***OsNHX1*** **and** ***OsNHX2*** **in the introgression line** ***RZ35*** **and its rice parental line,** ***Matsumae*** **.** The values shown are the means (± SE) of three biological replicates. Means followed by different letters among treatments at same time point are significantly different, according to least significant difference (LSD) test (*P* <0.05). 14-day-old seedlings were subjected to salt stress (100 mM NaCl) for 6 h, 24 h and 48 h.

Salt stress stimulated the expression of 5 genes of the salt overly sensitive (SOS) pathway: *OsSOS1*, *OsCIPK24*, *OsCBL4, OsNHX1* and *OsNHX2* and the Na^+^/H^+^ exchanger (NHX) gene families known to be involved in salt-tress response in shoots and roots in rice (Figures 5 and 6). Under salt stress, the steady-state transcript levels of *OsSOS1*, *OsCIPK24*, *OsCBL4* and *OsNHX2* in the shoots of *RZ35* were significantly lower than those in *Matsumae* at 6 h. The expression level of *OsNHX1* in shoots of *RZ35* was higher than that in *Matsumae* at 48 h under salt stress (Figure 6A, *P*<0.05). The expression levels of *OsSOS1*, *OsCIPK24*, *OsCBL4, OsNHX1* and *OsNHX2* in roots of *RZ35* were lower than those of *Matsumae* under salt stress (Figures 5 and 6).

### Altered Expression of the *High Affinity K*^*+*^*Transporter (HKT)* Gene Family

**Figure 7:**
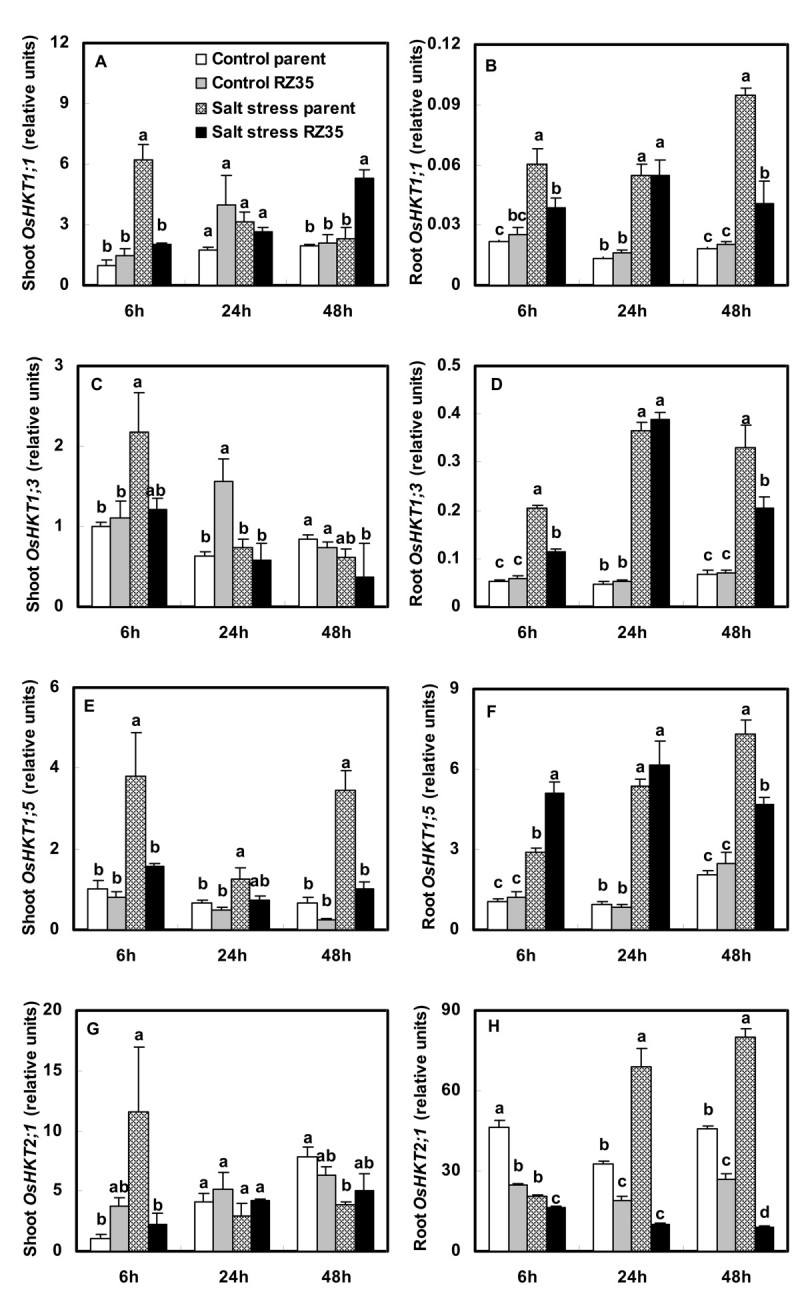
**Effects of salt stress on the expressions of** ***OsHKT1;1*** **,** ***OsHKT1;3*** **,** ***OsHKT1;5*** **and** ***OsHKT2;1*** **in rice-** ***Z. latifolia*** **introgressant (** ***RZ35*** **) and its rice parental line (** ***Matsumae*** **).** The values shown are the means (± SE) of three biological replicates. Means followed by different letters among treatments at same time point are significantly different, according to least significant difference (LSD) test ( *P* <0.05). 14-day-old seedlings were subjected to salt stress (100 mM NaCl) for 6 h, 24 h and 48 h.

Salt stress increased the steady-state transcript levels of *OsHKT1;1*, *OsHKT1;3* and *OsHKT1;5* in roots of both lines. Salt stress increased the expression levels of *OsHKT2;1* in roots of *Matsumae*, and decreased its expression level in roots of *RZ35*. Under salt stress, the expression levels of *OsHKT1;1*, *OsHKT1;3* and *OsHKT2;1* in roots of *RZ35* were lower than *Matsumae* (Figure 7, *P*<0.05). The expression levels of *OsHKT1;5* in shoots at all three time-points and at 48 h in roots of *RZ35* were lower than *Matsumae* (Figure 7).

### Altered Expression of the *Low Affinity K*^*+*^*Transporter 1 (AKT1)* and *KUP/HAK/KT K*^*+*^*Transporter (HAK)* Gene Families

**Figure 8:**
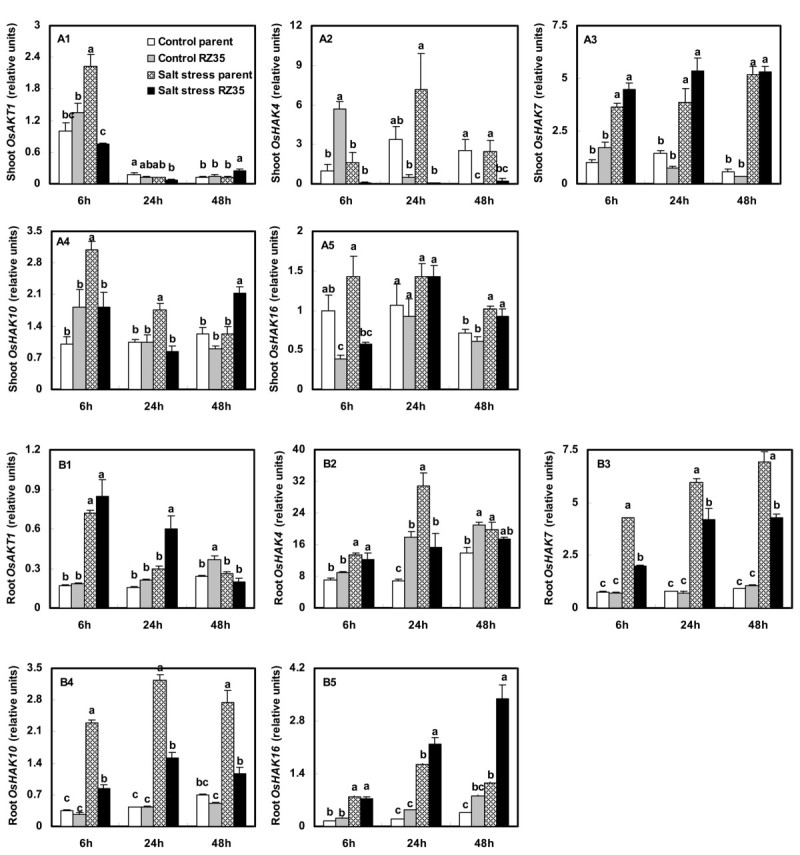
**Effects of salt stress on the expression of** ***OsHAK*** **family members and** ***OsAKT1*** **in rice-** ***Z. latifolia*** **introgressant (** ***RZ35*** **) and its rice parental line (** ***Matsumae*** **).** The values shown are the means (± SE) of three biological replicates. Means followed by different letters among treatments at same time point are significantly different, according to least significant difference (LSD) test (*P* <0.05). 14-day-old seedlings were subjected to salt stress (100 mM NaCl) for 6 h, 24 h and 48 h.

Under salt stress, the steady-state transcript level of *OsAKT1* in shoots of *RZ35* was lower than that in *Matsumae* at 6 h. The expression level of *OsAKT1* in roots of *RZ35* was higher than that in *Matsumae* at 6 h and 24 h (Figure 8, *P*<0.05). Salt stress strongly stimulated the expression of all members of the *OsHAK* family in roots of both lines. Under salt stress, the expression levels of *OsHAK7* and *OsHAK10* in roots of *RZ35* were lower than those in *Matsumae*, and the expression level of *OsHAK16* in roots of *RZ35* was higher than that of *Matsumae* at 24 h and 48 h (Figure 8, *P*<0.05).

## Discussion

That introgression could enhance stress tolerance or adaptability has been documented by numerous studies. For example, the introgression from a wild rice species (*Oryza rufipogon* Griff.) increased the drought tolerance of the recipient rice cultivar (Zhang et al. 2006). The introgression from *Thinopyrum ponticum* (Chen et al. 2004) and *Th. hessarahicum* (King et al. 1997) improved salt-tolerance of wheat, and the introgression of the *PcINO1* gene from *Porteresia coarctata* conferred salt tolerance to tobacco plants (Das-Chatterjee et al. 2006). The introgression from wild rice species increased yield of rice under drought condition (Bimpong et al. 2011; Kato et al. 2011). The introgression of SUB1, a major submergence-tolerance QTL, enhanced the yield of the recipient rice cultivar under submergence condition (Singh et al. 2009). It should be pointed out that in all these examples, the altered traits could be explained by either direct transfer and/or allelic or non-allelic genic interactions (e.g., epistasis) between the two parental species. In contrast, in our case, because the amount of genetic introgression from *Zizania latifolia* is minute on one hand, and extensive genetic and epigenetic *de novo* variations occurred on the other (Liu et al. 2004; Shan et al. 2005; Wang et al. 2005), it is most likely that the altered trait (salt tolerance) occurred *de novo*. Also, in contrast with the above examples, we observed the trait alteration in an opposite direction (Figures 1 and 2), that is, the introgression by *Z. latifolia* has reduced salt tolerance in the introgression line (*RZ35*) compared with its rice parental line (*Matsumae*).

Lower Na^+^ and higher K^+^ in the cytoplasm are essential for maintaining kinetic activity of a number of enzymes (Munns and Tester 2008). Na^+^ enters plant cells principally through K^+^ pathways (Blumwald 2000). The similarity of the hydrated ionic radii of Na^+^ and K^+^ make them difficult to be discriminated from each other, and this constitutes the basis of Na^+^ toxicity (Blumwald 2000). Na^+^, Cl^-^ and Na^+^/K^+^ are important physiological selection criteria for salt tolerance in plants (Ashraf 2004). Our results indicated that, when plants were subjected to salt stress for 48 h, the Na^+^, Cl^-^ and Na^+^/K^+^ ratio in shoots of the introgressant (*RZ35*) were higher than those of its parental line (*Matsumae*), while their values in roots of the introgressant were lower than those in the rice parental line (Figures 3 and 4). This suggested that the introgression has strongly influenced the ion accumulation of the recipient rice under the salt stress condition (Figures 3 and 4), and escalated the injuries of Na^+^ and Cl^-^ in shoots. This might be the major cause for the damage of salt stress on the introgressant *RZ35* at the physiological level, which was much more severe than that of the parental line. We suspected that the larger amount of accumulation of Na^+^ in rice shoots induced by the introgression might be related to the expression alteration of genes related to Na^+^/K^+^ balance.

Indeed, We found that salt stress strongly stimulated the expression of the *OsSOS1*, *OsCIPK24*, *OsCBL4, OsNHX* and *OsHKT* gene families in rice (Figures 5, 6, 7). These genes have been shown to play vital roles in conditioning salt tolerance in rice (Negrão et al. 2011). Relative to its rice line (*Matsumae*), the introgressant (*RZ35*) showed significantly lower levels of expression of *OsSOS1*, *OsCIPK24*, and *OsCBL4* in both shoots and roots under salt stress (Figure 5). Also, the introgressant (*RZ35*) showed lower expression levels of *OsNHX2, OsHKT1;1*, *OsHKT1;3* and *OsHKT2;1* in roots, and lower expression level of *OsHKT1;5* in both shoots and roots (at 48 h) than *Matsumae* under salt stress (Figures 6 and 7). It was widely recognized that *OsSOS1* played important roles in Na^+^ exclusion from roots into the rhizosphere; *OsSOS1* and *OsHKT1;5* played important roles in the control of long-distance transport from roots to shoots and contributed to Na^+^ exclusion from shoots to the roots (Shi et al. 2002; Ren et al. 2005; Munns and Tester 2008; Horie et al. 2009). For example, Shi et al. (2002) clearly demonstrated that *SOS1* was critical for controlling long-distance Na^+^ transport from root to shoot, and *SOS1* functioned in retrieving Na^+^ from the xylem stream under severe salt stress (Shi et al. 2002). Ren et al. (2005) also showed that OsHKT1;5 mediates Na^+^ unloading from xylem vessels at the plasma membrane of xylem parenchyma cells, namely OsHKT1;5 mediated sodium exclusion from leaves or shoots (Ren et al., 2005), which has been widely accepted (Ren et al. 2005; Negrão et al. 2011). The down-regulation of *SOS1*, *CIPK24*, *CBL4* and *OsHKT1;5* may have increased Na^+^ content in shoots because their down-regulation decreased the frequency of Na^+^ exclusion from shoots or roots (Ren et al. 2005; Munns and Tester 2008; Negrão et al. 2011). Together, these data strongly suggested that, under salt stress, the introgression increased the Na^+^ content in shoots of of the introgression line via down-regulation of *OsSOS1*, *OsCIPK24* , *OsCBL4* and *OsHKT1;5*. This suggested that the introgression modified the capacity of the rice roots to control the transportation of Na^+^ from roots to shoots via a gene expression regulation. This conclusion was also supported by the ion selective transport capacity (ST) results. The ST value of the introgressant was lower than that of the rice parental line at 48 h under salt stress (Figure 3G). Our results may explain why the Na^+^ content in shoots of the introgressant was higher than that of the rice parental line and why in roots of the introgressant it was lower than that in the parental line under salt stress (Figure 3). Furthermore, the introgression also greatly affected the K^+^ metabolism (Figure 3C and D). Trans-membrane K^+^ movements in plants are mediated by several types of channels, including the AKT family, and by transporters that belong to two families, KcsA-TRK (HKT) and KUP/HAK/KT (HAK) (Bañuelos et al. 2002; Amrutha et al. 2007). Figure 8 shows that the introgression strongly affected the expression of the *OsHAK* gene family and *OsAKT1*. The expression levels of *OsHAK7* and *OsHAK10* in roots of the introgressant were lower than those of the parental rice line under salt stress, which might be useful to the K^+^ supply of introgressant shoots.

In summary, our results showed that the introgression from *Z. latifolia* affected growth, salt stress response and metabolism of the recipient rice cultivar. It will be interesting to elucidate by what means the introgression process (rather than the introgression *per se*) would have concomitantly affected numerous metabolic pathways or links such as K^+^ metabolism (changed the expressions of *OsHAK* family and *OsAKT1*), Na^+^ control, protein kinase (*OsCIPK24*), and Ca^2+^ binding protein (*OsCBL4*)*.* One conceivable possibility is the extensive epigenetic remodeling in the form of altered DNA methylation that occurred in many genomic loci in this introgression line is responsible for the down-regulated expression of these analyzed genes under the salt tress condition (Liu et al. 2004). It has been established that altered DNA methylation at critical regions of a master regulatory gene may exert profound effects on the expression of many downstream target genes (Lukens and Zhan 2007; Chinnusamy and Zhu 2009; Doerfler 2011). An epigenetic underpin may also explain the differential effect by the introgression on gene expression in different tissues, e.g., leaf vs. root of *RZ35*. Further studies are needed to investigate this possibility.

## Conclusions

In summary, our results showed that the introgression from *Z. latifolia* affected growth, salt stress response and metabolism of the recipient rice cultivar. Contrary to our expectation, the results showed that the inhibitory effect of salt stress on growth of *RZ35* was significantly greater than that of *Matsumae*. We further found that a major underlying cause for this outcome is that the introgression process weakened the capacity in Na^+^ exclusion under the salt stress condition, and hence, escalated the injuries of Na^+^ and Cl^-^ in shoots of *RZ35*. Accordingly, four genes known to be involved in the Na^+^ exclusion, i.e., *OsHKT1;5*, *OsSOS1*, *OsCIPK24* and *OsCBL4*, were found to be significantly down-regulated in roots of *RZ35* relative to its rice parental line under the salt stress condition, thus implicating a gene expression regulation-based molecular mechanism underlying the difference in salt stress-tolerance between the introgression line and its rice parental line.

## Methods

### Plant materials

The introgression line (*RZ35*) derived from a cross between rice (cv. *Matsumae*) and *Zizania latifolia* by a novel, simple approach called “repeated pollination” (Liu et al., 1999; Shan et al., 2005), was used in this study. This stabilized introgressant (at the 10th selfed generation) was homogeneous in phenotype and DNA fingerprinting patterns, and exhibited heritable, novel morphological characteristics in multiple traits compared with its rice parental cultivar *Matsumae* (Shan et al. 2005; Wang et al. 2005).

### Plant growth conditions

Seeds of the introgressant (*RZ35*) and its rice parental line *Matsumae* were germinated in petri dishes for 6 d in a growth cabinet (28°C during the day and 24°C during the night, 16/8 h photoperiod at 50 μmol m^–2^ s^–1^). The seedlings were then transferred to barrels containing 2000 mL of aerated sterile nutrient solution under hydroponic culture, and the nutrient solution was replaced every two days. The barrels were placed in a growth chamber that was maintained at 27.0 ± 1°C during the day and 22.0 ± 1°C during the night, under a 16/8 h photoperiod at 250 μmol m^–2^ s^–1^. The nutrient solution used in this work contained 0.715 mM NH_4_NO_3_, 0.16 mM NaH_2_PO_4_, 0.323 mM K_2_SO_4_, 0.5 mM CaCl_2_, 0.83 mM MgSO_4_, 0.036 mM Fe-EDTA, 0.1 mM Na_2_SiO_3_, 4.55 μM MnCl_2_, 0.077 μM ZnSO_4_, 0.078 μM CuSO_4_, 9.25 μM H_3_BO_3_, 0.263 μM H_2_MoO_4_, and pH = 5.2.

### Stress treatment

After 10 days of growth in hydroponic culture medium, rice plants were subjected to salt stress (100 mM NaCl) by transferring them to another barrel containing 2000 mL of the treatment solution amended with the above nutrients and 100 mM NaCl. A barrel including 20 seedlings represented one replicate. For each line, 24 barrels of seedlings were randomly divided into 8 sets, three buckets per set. Each barrel was considered as one replicate with three replicates per set. Four sets were used as control (one set per time point), and the remaining four sets were treated with salt stress. Treatment solutions were replaced daily. The nutrient solution without stress salts was used as a control. The seedlings were harvested after treatment for 6, 24, and 48 h respectively. When plants were subjected to salt stress for 5d, the plants were photographed to test tolerance of introgressant (*RZ35*) and its rice parental (cv. *Matsumae*) to salt stress. Then the electrolyte leakage rate was determined with the ameliorated method of Lutts et al. (1996). One fresh whole plant (shoot) from each barrel was washed three times with deionized water to remove surface adhered electrolytes, then was placed in a closed cuvette containing 20 mL of deionized water at 25°C for 6 h. The electrical conductivity of the solution (EC1) was determined with a conductivity gauge. After this the cuvette was autoclaved at 100°C for 20 min, and the electrical conductivity of the solution (EC2) was determined. Electrolyte leakage rate can be defined as follows: Electrolyte leakage rate (%) = (EC1/EC2) × 100. Remaining plant materials in each barrel were immediately frozen in liquid nitrogen and then stored at −70°C for malondialdehyde (MDA) determination.

### Measurements of physiological indices

The roots and shoots of 10 seedlings in each barrel were immediately frozen in liquid nitrogen and then stored at −70°C for RNA isolation. Another 10 seedlings in each barrel were washed with distilled water, after which the roots and shoots were separated and lyophilized. Then all dry samples of 10 seedlings in each barrel were levigated and mixed for ion measurements. The dried samples of the roots and shoots were digested with HNO_3_, and the Na^+^ and K^+^ contents were assayed using an atomic absorption spectrophotometer (TAS-990, Purkinje General, Beijing). Dry samples of plant material were treated with 10 mL deionized water at 100°C for 2 h, and the extract was then used to determine the Cl^–^ content. The Cl^–^ content was determined by ion chromatography (DX-300 ion chromatographic system; AS4A-SC ion-exchange column, CD M-II electrical conductivity detector, mobile phase: Na_2_CO_3_/NaHCO_3_ = 1.7/1.8 mM; DIONEX, Sunnyvale, USA). MDA content was determined with the method of Zhao et al. (1994).

### Quantitative real-time reverse-transcription (RT)-PCR analysis

Total RNA was extracted from the shoots and roots of seedlings grown under the salt-stress or non-stress conditions using TRIzol reagent (Invitrogen). The RNA was treated with DNaseI (Invitrogen), reverse-transcribed using SuperScriptTM RNase H-Reverse Transcriptase (Invitrogen), and then subjected to real time PCR analysis using gene-specific primers. The gene-specific primers are listed in Table 1. PCR amplification was conducted with an initial step at 95°C for 1 min followed by 40 cycles of 5 s at 95°C, 10 s at 60°C and 30 s at 72°C. Amplification of the target gene was monitored every cycle by SYBR Green. Amplification of the rice *βactin* (GenBank Accession AK101613) mRNA was used as an internal quantitative control (Sripinyowanich et al. 2010; Wang et al. 2010). PCR reaction conditions were optimized, after which the amplification efficiencies of each target gene and reference gene were approximately equal. The relative expression of the target genes was measured using the comparative Ct method (Livak and Schmittgen 2001). Real time PCR analyses were conducted at least three times for each sample.

**Table 1:** **Gene-specific primers used in real time quantitative RT-PCR analysis**

Gene name	GenBank Accession No.	Forward primer (5′-3′)	Reverse primer (5′-3′)
*OsNHX1*	AB021878	GTTCAAGAGTTACAACAAAGCACG	CAGCGGGAATACAAAAGCAG
*OsNHX2*	AY360145	ACCAAGACGAAACACCCCTAC	AACCCAGCAACTACTCCAAGAA
*OsHKT1;1*	AJ491816	ATTAGCAGAGCACTGTGGAGGAA	CCGACGAACCCGTAGGAAG
*OsHKT1;3*	*AJ491818*	*CAGTTCATCTACCAAAACAATCCA*	*AATACCTCACCACCAATCAGCA*
*OsHKT1;5*	DQ148410	TGCCACCTTACACCACTTTCG	TGCCATACGCACTGATAACCTC
*OsHKT2;1*	AB061311	GCATATTCACCCATTCTGGATTCAGT	CGATGGTGATGAGGCTGGAAAGT
*OsSOS1*	AY785147	CTCCGTGCTCATAGAATCGC	ATACTCACTCAAGTGGGTCAATACC
*OsCBL4*	AK101368	GGCATCGTTCGGATTTCAC	GAGATTCGCCTTTCTGCTGTT
*OsCIPK24*	AK102270	AAGAAGCGGGTGGGGAGGT	GCGGTGGTTGAGGATGGTGT
*OsAKT1*	AY065970	TACGACCGCCGATACAGAA	CCAAATAAGCCACAAAGAAGG
*OsHAK4*	AF129485	CGTTCCCATCCGTCAGTAAA	CAGCCTCTGGTCTGGTTCGTC
*OsHAK7*	AJ427971	GAACTCCAACTTCCTCAAGACG	AGATCATGCCGACTTCGACGAG
*OsHAK10*	AJ427972	CGCTCTCGGCTGCTTTCCT	TAACCGCCAATCCTGACGC
*OsHAK16*	AJ427973	AGCGACTGTGTGCTAAACCC	CATAGATGCCAATCCCTGAGA
*β*-actin	AK101613	ATGCCATTCTCCGTCTT	GCTCCTGCTCGTAGTC

NHX, Na^+^/H^+^ exchanger; HKT, high affinity K^+^ transporter; HAK, KUP/HAK/KT K^+^ transporter; AKT, low affinity K^+^ transporter; SOS, salt overly sensitive.

### Calculation of ion selective transport capacity

Values of the selective transport capacity (ST*)* by different plant parts for K^+^ over Na^+^ were estimated following the equation of Wang et al. (2002) : ST (root/shoot) = (Na^+^/K^+^ in root)/(Na^+^/K^+^ in shoot). The bigger the ST value, the stronger the root controls Na^+^ and promotes transport of K^+^ to shoot, indicating a stronger selective transport capacity of the root.

### Survival rate

The seeds of both rice lines were germinated and grown in petri dishes for 6 d in a growth cabinet (28°C during the day and 24°C during the night, 16/8 h photoperiod at 50 μmol m^–2^ s^–1^). The seedlings were cultured and stressed using the above methods. After 10 days of growth in hydroponic medium, rice plants were subjected to stresses by transferring them to another barrel containing 2000 mL of the treatment solution amended with the above nutrients and 100 mM NaCl. A barrel including 50 seedlings represented one replicate, and there were three replicates per treatment. For each rice line, 6 barrels of seedlings were randomly divided into 2 sets, three barrels per set. Each barrel was considered as one replicate with three replicates per set, one set was used as control, and another set was treated with salt stress. The nutrient solution without stress salts was used as a control. The survival rates were calculated after treatment for 13 d.

### Statistical analysis

Statistical analysis of the data was performed using the statistical program SPSS 13.0 (SPSS, Chicago, USA). All data were represented by an average of the three biological replicates and the standard errors (S.E.). The treatment mean values at same time point were compared by post hoc least significant difference (LSD) test. The term significant indicates differences at *P* <0.05.

## Authors’ original submitted files for images

Below are the links to the authors’ original submitted files for images. Authors’ original file for figure 1 Authors’ original file for figure 2 Authors’ original file for figure 3 Authors’ original file for figure 4 Authors’ original file for figure 5 Authors’ original file for figure 6 Authors’ original file for figure 7 Authors’ original file for figure 8

## Abbreviations

SOS: Salt overly sensitive
NHX: Na^+^/H^+^ exchanger
HKT: High affinity K^+^ transporter
HAK: KUP/HAK/KT K^+^ transporter
AKT: Low affinity K^+^ transporter
ST: Selective transport for controlling Na^+^ and promoting transport of K^+^.
