# Supplementary figures and images for: Heritable alteration in salt-tolerance in rice induced by introgression from wild rice (Zizania latifolia)
Source: Rice (N Y). 2012 Dec 19;5:36. doi: 10.1186/1939-8433-5-36 (PMC4883729; doi:10.1186/1939-8433-5-36)

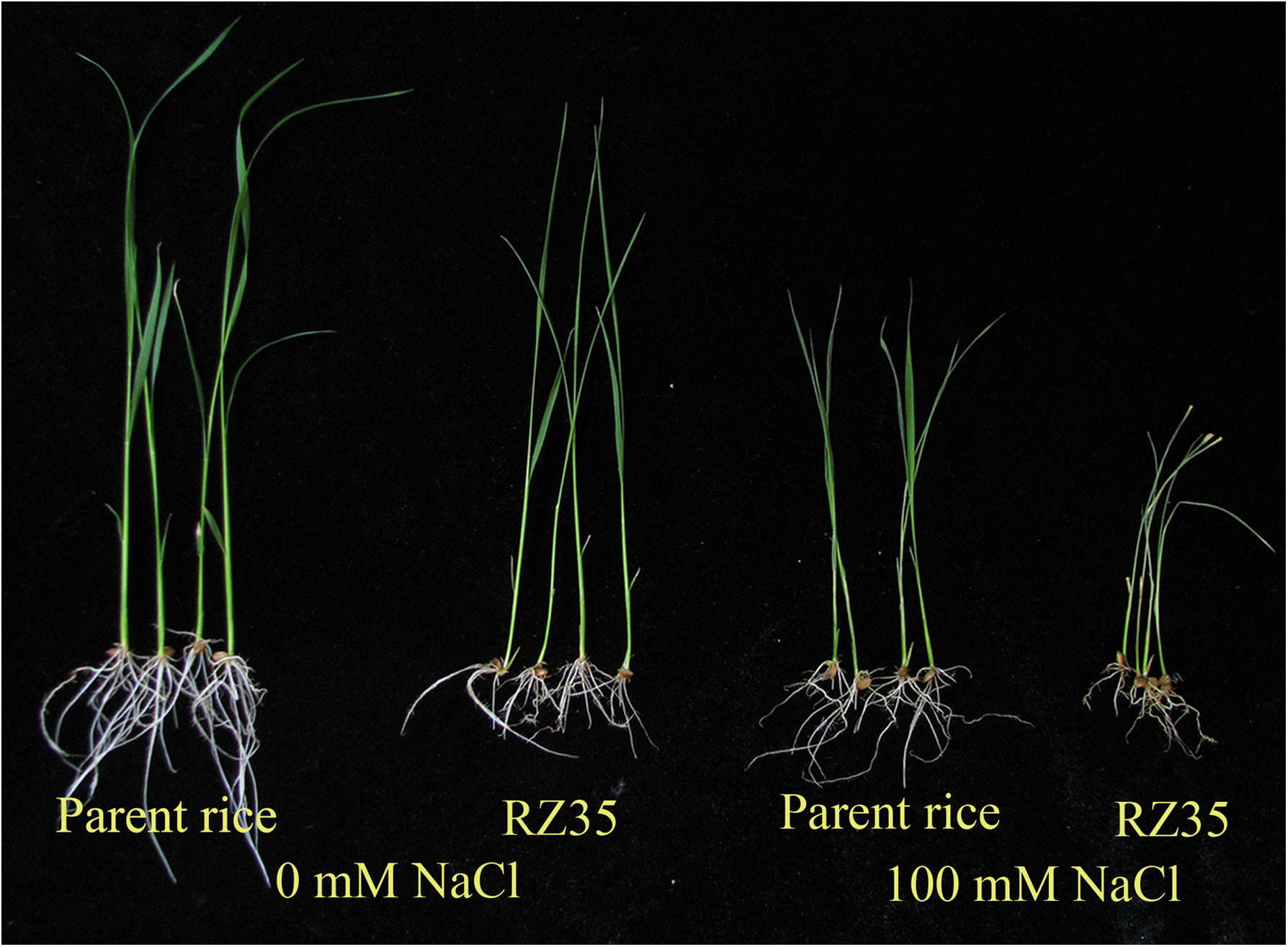

Supplement: Supplementary file 1 — Authors’ original file for figure 1 [file 12284_2012_35_MOESM1_ESM.tiff]

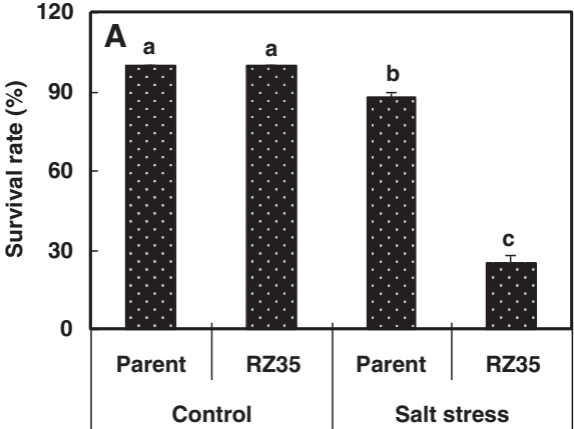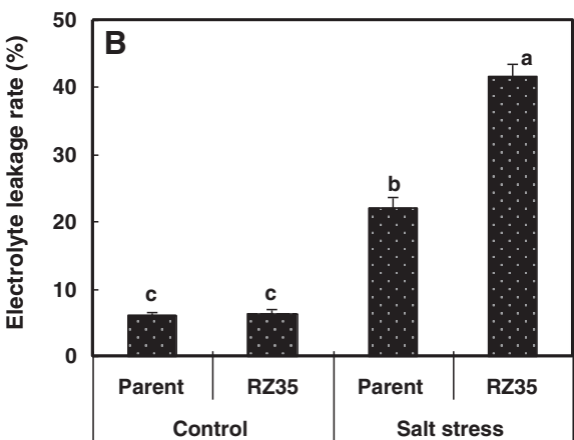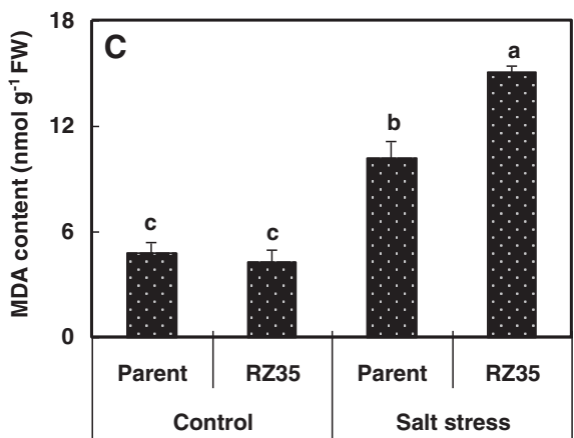

Supplement: Supplementary file 2 — Authors’ original file for figure 2 [file 12284_2012_35_MOESM2_ESM.pdf]

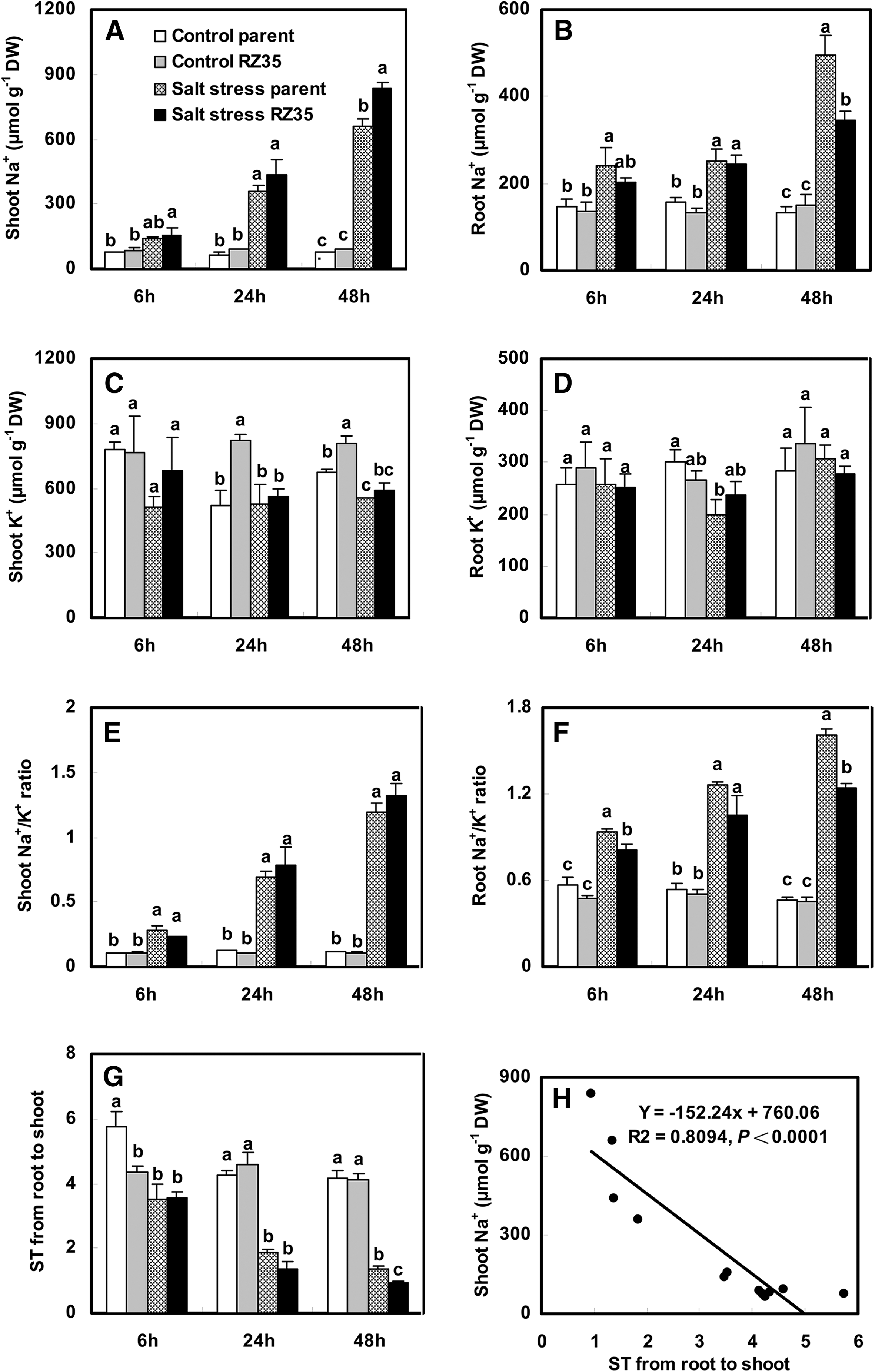

Supplement: Supplementary file 3 — Authors’ original file for figure 3 [file 12284_2012_35_MOESM3_ESM.tiff]

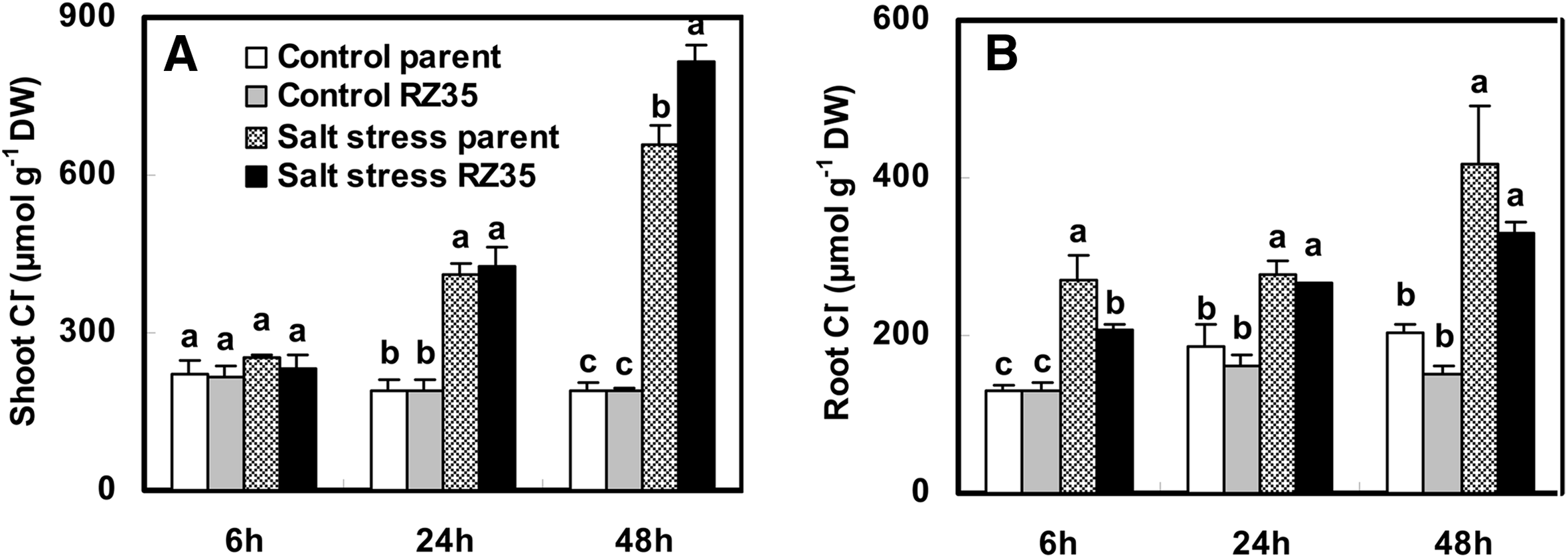

Supplement: Supplementary file 4 — Authors’ original file for figure 4 [file 12284_2012_35_MOESM4_ESM.tiff]

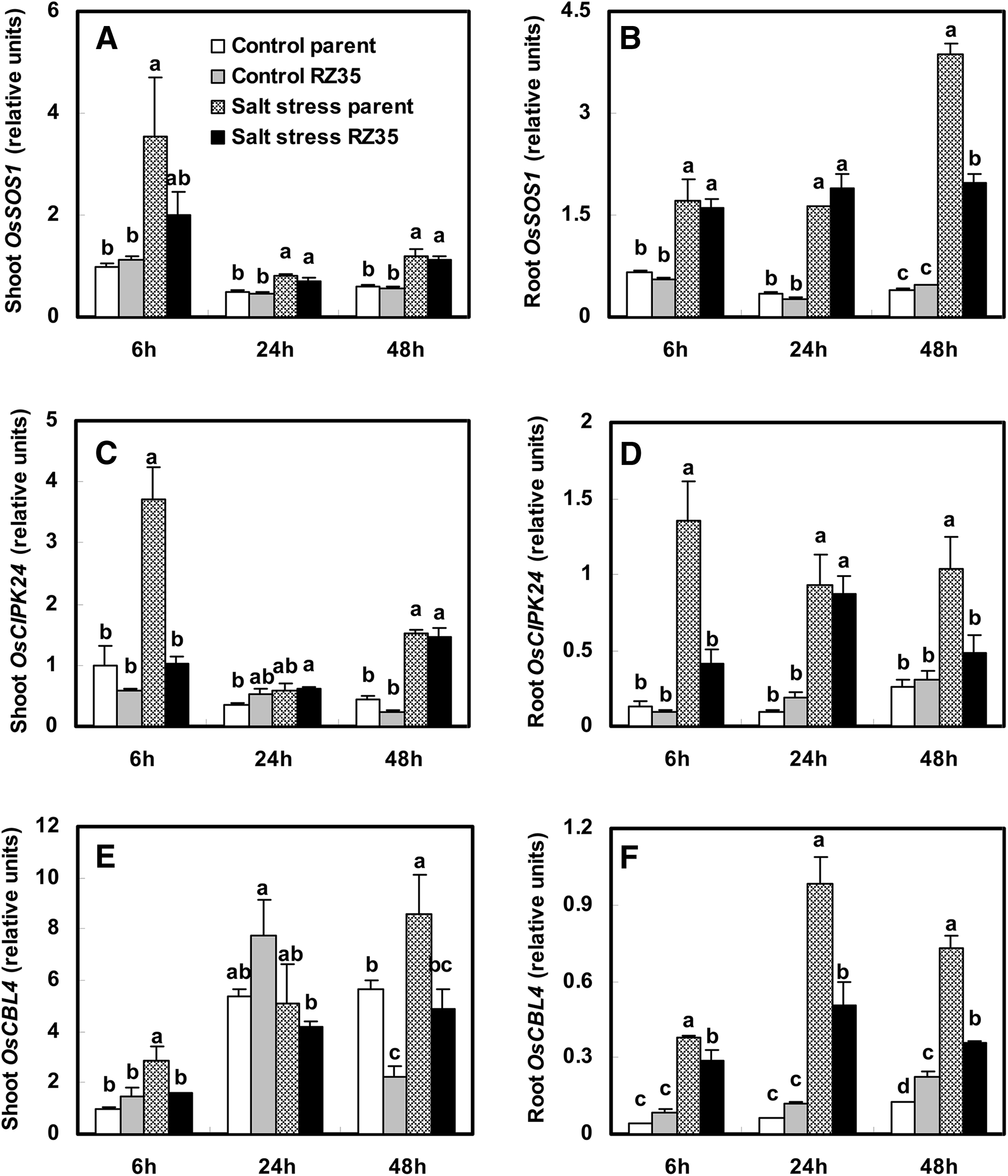

Supplement: Supplementary file 5 — Authors’ original file for figure 5 [file 12284_2012_35_MOESM5_ESM.tiff]

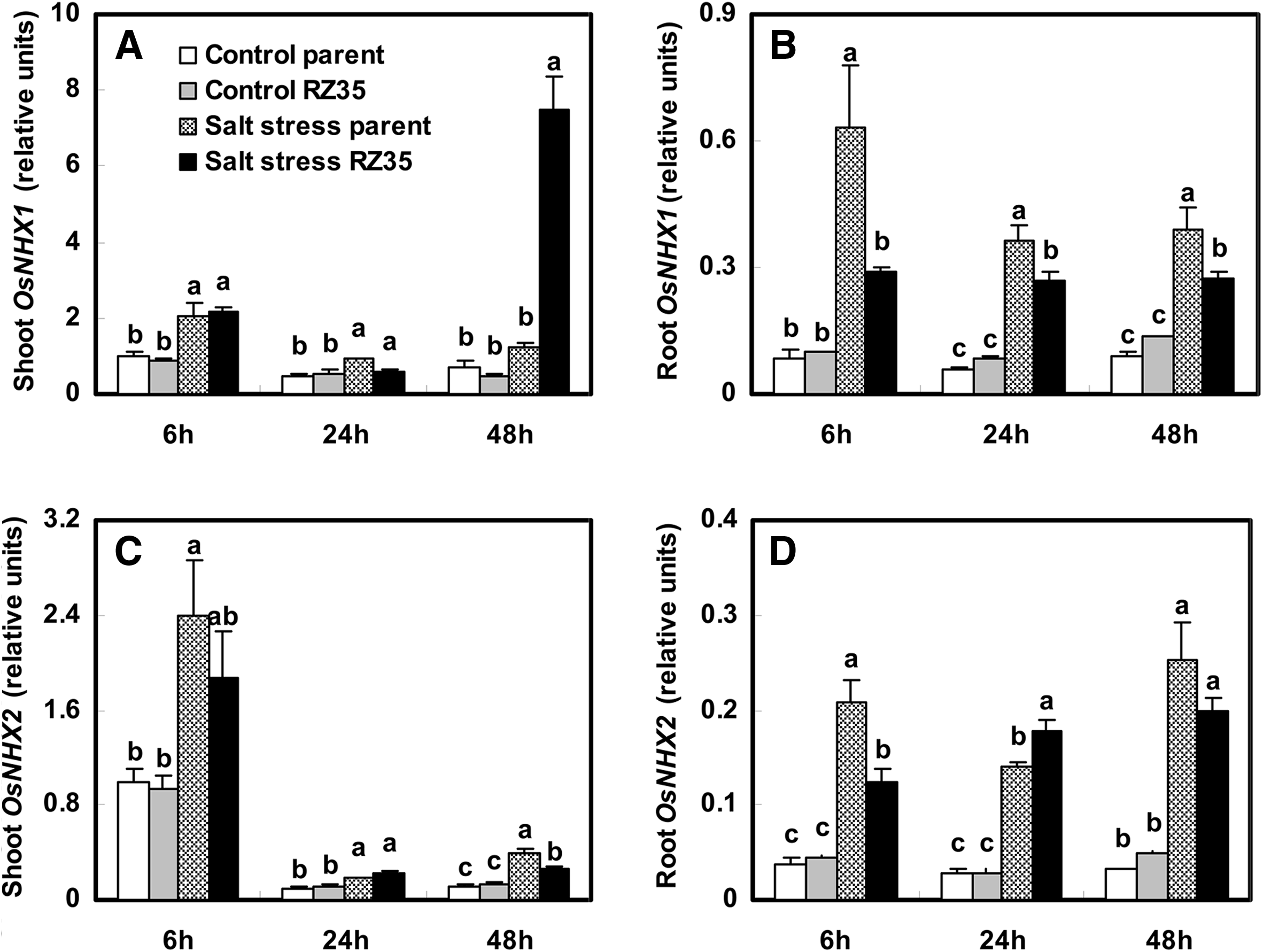

Supplement: Supplementary file 6 — Authors’ original file for figure 6 [file 12284_2012_35_MOESM6_ESM.tiff]

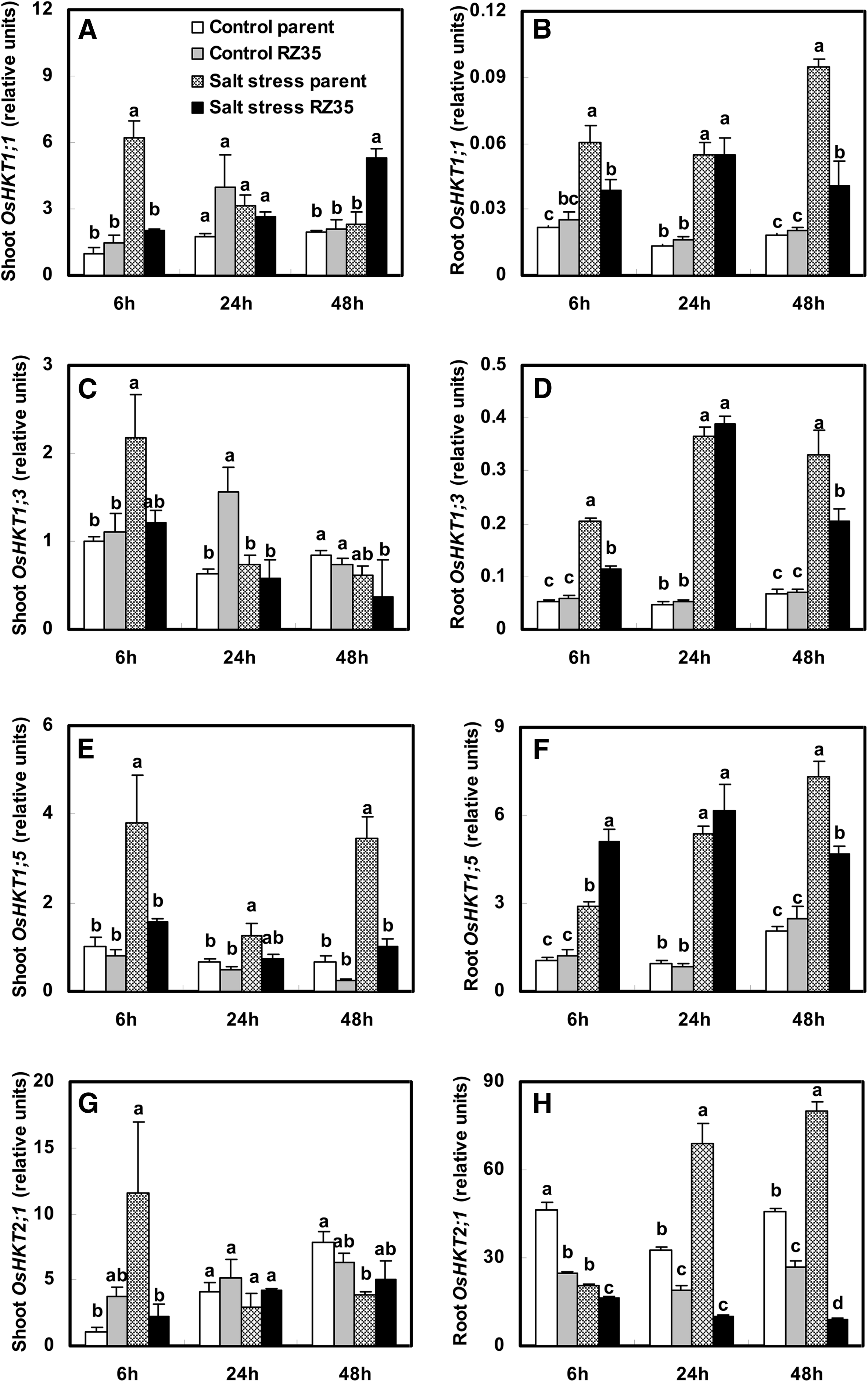

Supplement: Supplementary file 7 — Authors’ original file for figure 7 [file 12284_2012_35_MOESM7_ESM.tiff]

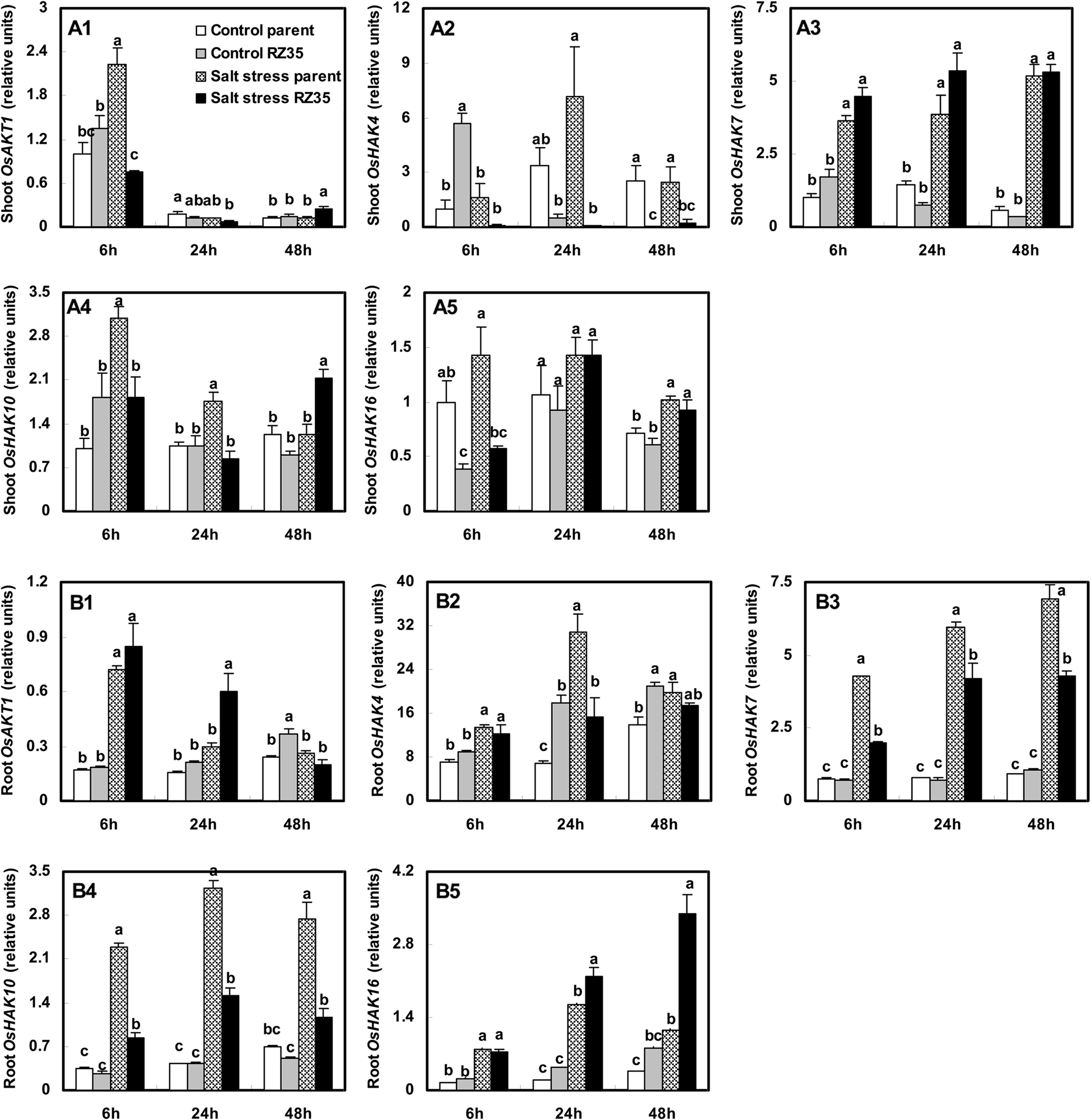

Supplement: Supplementary file 8 — Authors’ original file for figure 8 [file 12284_2012_35_MOESM8_ESM.tiff]
